# Assortative Mating and Increase in Prevalence and Severity of Autistic Spectrum Disorder in Children—A Systematic Review

**DOI:** 10.3390/children13020244

**Published:** 2026-02-09

**Authors:** Michael Eisenhut, Anjana Jeevan

**Affiliations:** Paediatric Department, Luton & Dunstable University Hospital, Lewsey Road, Luton LU4 0DZ, UK; anjana.jeevan.20@ucl.ac.uk

**Keywords:** autism, autistic spectrum disorder, assortative mating, protective alleles, non-random mating

## Abstract

**Highlights:**

**What are the main findings?**

- Assortative mating is the likely cause of the increase in prevalence of autistic spectrum disorders.
- In countries with assortative mating, the prevalence of autistic spectrum disorders is higher.

**What are the implications of the main findings?**

- There is an urgent need to investigate mating of which phenotypical features and which genes contributing to an autistic spectrum disorder phenotype are associated with learning difficulties in the offspring.
- Future studies need to investigate which genetic constellations in spouses if combined in an offspring lead to manifestations of more severe autistic spectrum disorder.

**Abstract:**

**Background/objectives:** The prevalence of autistic spectrum disorder has been increasing rapidly in the world population and the cause of this increase is unknown. Autistic spectrum disorder is an important cause of social, communication and specific learning difficulties in children. Assortative mating may increase the genetic burden leading to manifestation of polygenic diseases affecting mental health in the offspring. Correlation of scores in the social responsiveness scale (SRS), which is used to quantify autistic spectrum disorder features, between spouses, has been used as indicator of phenotypic assortative mating. We investigated whether assortative mating is involved in increased severity of autism spectrum disorder in the offspring. **Methods:** All studies reporting on investigation of assortative mating in relationship to autistic spectrum disorder were included. Information sources were PubMed, EMBASE and the Cochrane Library. Results were synthesized by entering correlation analyses of results of the SRS conducted in spouses in a meta-analysis. A sub-group analysis was performed comparing spouses with offspring with diagnosed autistic spectrum disorder to spouses without. Prevalence of autistic spectrum disorders in children in countries with and without predominant assortative mating was compared. **Results**: A total of 14 investigations of assortative mating including 9914 spouse pairs were included. In total, 8 studies (4641 spouse pairs) reported intra-class correlation (ICC) or Spearman’s correlation coefficients between spouses’ SRS scores. There was a significant correlation of SRS scores in studies using ICC or Spearman’s correlation with a pooled coefficient = 0.37. Spouse pairs (n = 401) with offspring diagnosed with autistic spectrum disorder had a pooled ICC coefficient which was 0.278 (95% CI 0.08 to 0.46), significantly lower than spouse pairs without (n = 1525): 0.40 (95% CI 0.35 to 0.46). Higher scores in SRS of both spouses were associated with higher scores and more autism diagnoses in offspring. Pooled prevalence of autistic spectrum disorder in children in countries where assortative mating is most common was 63.1 per 10,000 of population and in countries without it was significantly lower with 14.1 per 10,000 of population. **Conclusions**: There is evidence of assortative mating according to social responsiveness scale score which correlates significantly in spouse pairs with and without children with autistic spectrum disorder. In countries where assortative mating is predominant, a higher prevalence of autism spectrum disorder in children is found compared to countries without.

## 1. Introduction

### 1.1. Rationale

Autism spectrum disorder (ASD) is an umbrella term for a group of neurocognitive phenotypes which have as core features social communication deficits and restricted or repetitive sensory–motor behaviors manifesting, for example, in repetitive hand or finger movements or handling of objects, and repetitive use of phrases, interests or activities which are different from the majority of the population. Up to 2.3% of children are currently affected by ASD [1,2]. According to a recent systematic review, overall, 33% of people with ASD have been reported as having learning difficulties linked to this disorder [3]. The cause is unknown. It cannot be environmental factors because twin studies showed that 92% of monozygotic twin pairs were concordant for a broader spectrum of related cognitive or social abnormalities versus 10% of dizygotic twin pairs. This was shown in a British study of forty-four sets of twins and triplets, which were available for genetic analysis and these contained 59 autistic individuals [4], demonstrating that this condition is inherited. The largest previous systematic review into environmental risk factors identified metabolic syndrome features in pregnancy, which are related to obesity, as the only convincingly associated factor, which may also explain the association with testosterone exposure [5]. This association could be due to the fact that undiagnosed masked autistic spectrum disorder gene expression in the pregnant women included caused obesity in pregnancy and, at the same time, autism spectrum disorder in the offspring. Autistic spectrum disorder is associated with increased BMI in 33% [3] of individuals; the largest systematic review on the association of obesity with autism revealed, from the analysis of fifteen studies encompassing 49,937,078 participants and 1,045,538 individuals with ASD, that the prevalence of obesity was significantly higher in individuals with ASD than in controls (OR  =  1.84, 95% confidence interval [CI]: 1.37–2.48, *p*  <  0.001) [6]. The global increase in obesity prevalence is therefore unlikely to be the cause of an increase in autism spectrum disorder prevalence. Global epidemiological studies have demonstrated that the prevalence of autism spectrum disorders is increasing world-wide [2], with a doubling of prevalence in the USA from 2008 to 2018 from 1.1 to 2.3%; in South Korea from 5.04 (per 100,000) in 2008, to 10.97 in 2015 [7]; and in Taiwan [8], Hong Kong [9], Thailand [10] and France with a significant increase between 1997 and 2003 [11]. The question therefore arises as to how an overwhelmingly genetic condition can increase rapidly in the general population without it being simply a reflection of increased awareness or diagnostic capability.

Assortative mating is a non-random mating system which occurs when there is a correlation (positive or negative) between male and female phenotypes or genotypes across mated pairs. Assortative mating can be measured as a correlation between the values of homologous phenotypic or genotypic traits across members of mated pairs. Assortative mating may be either positive, implying a tendency to mate with phenotypically similar individuals, or negative (also called dis-assortative), implying the converse [12]. When the trait has a genetic component and assortative mating occurs, both genetic and phenotypic assortative mating take place. Assortative mating can thereby increase in the offspring the deviation of a phenotype from the average. This will then cause a phenotype in the offspring with enough features to enable a diagnosis and hence increase the prevalence of a condition being diagnosed in the general population. Assortative mating has first been proposed as a mechanism for increase in autism spectrum disorder prevalence by Professor Sir Simon Baron-Cohen based on his observation of similarities in spousal cognitive phenotype amongst parents of children with autistic spectrum disorder [13,14].

The goal of this systematic review is to investigate whether assortative mating is involved in the increase in prevalence of autism spectrum disorders in the offspring by parents who have chosen each other on the basis of a phenotype resulting from genes involved in the pathogenesis of autistic spectrum disorder increasing the overall genetic load of autism spectrum disorder causing genes in the offspring. This increased genetic load would then cause the phenotype to be more severe and above the threshold enabling a diagnosis and hence increasing the prevalence of diagnosed cases. Assortative mating forms the basis of marriage in the United States of America, Mexico, South American countries, European countries, Israel, Lebanon, China, Taiwan and Australia [15]. Epidemiological investigations in Saudia Arabia, India, Bangladesh and Nepal revealed that the majority of marriages are arranged [16,17,18,19,20].

### 1.2. Objectives

- To investigate whether assortative mating guided by features of autistic spectrum disorder occurs.
- To investigate whether, if assortative mating occurs, there are specific personal characteristics guiding the mating more than others.
- To investigate whether assortative mating is involved in increased severity of autism spectrum disorder in the offspring.
- To investigate if autistic spectrum disorder is less common in countries where mating is not assortative compared to countries where assortative mating can occur due to individual partner choice.

## 2. Methods

The review was structured and conducted following the PRISMA 2020 guidelines: See check list in Appendix A.

### 2.1. Eligibility Criteria

Inclusion criteria

Included were all studies reporting on assortative mating of the following participants:

- Adults and children with a diagnosis of autistic spectrum disorder;
- Adults and children with phenotypic traits of an autistic spectrum disorder;
- Adults and children with genes associated with autistic spectrum disorder;
- Adults and children without the above phenotypic features, diagnoses or genes of autistic spectrum disorder used as control groups for the above groups.

For investigation of the influence of marriage practices based on assortative mating versus arranged marriages, population-based studies applying validated tools for diagnosis of autistic spectrum disorder to all participants were included. Only the most recent such investigation for each country was included. If there was more than one study from a country, only those conducted on non-overlapping populations were included.

Exclusion criteria

Studies where the criteria for diagnosis of autistic spectrum disorder or traits of autistic spectrum disorder were not documented or assortative mating was not investigated were not included.

### 2.2. Information Sources

The search strategy was as follows: Both authors searched the databases PubMed (from inception in January 1996), EMBASE (from inception in 1974) and the Cochrane library (from inception in April 1996) using the following keywords: (autism OR autistic spectrum disorder OR autism spectrum disorder) AND (assortative mating OR random mating OR non-random mating OR protective alleles). In addition, literature quoted was retrieved and screened for eligibility. Data on prevalence of autistic spectrum disorders were obtained from already existing recent systematic reviews [21,22,23].

**Figure 1:**
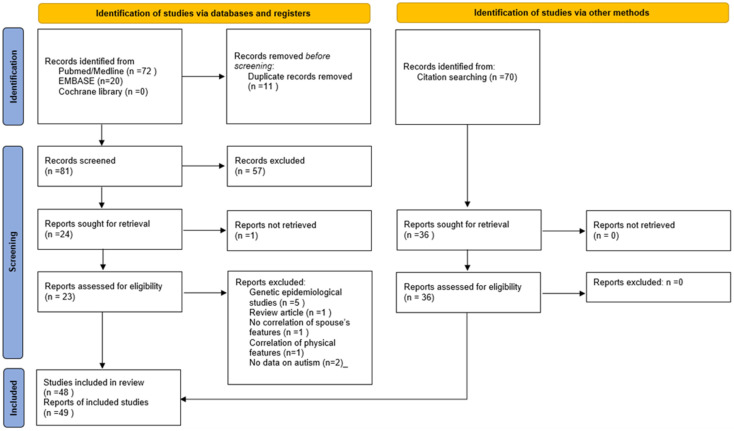
PRISMA 2020 flow diagram for new systematic reviews which included searches of databases, registers and other sources. Source: [24].

The selection process was documented following the PRISMA 2020 guidance [24] (see Figure 1).

The data collection process was performed using an excel word spreadsheet.

Data items extracted included characteristics of the population of each study, number of spouse pairs, tool used for ascertainment of features of autistic spectrum disorder, statistical tool used for ascertainment of assortative mating and result of correlation analyses.

### 2.3. Study Risk of Bias Assessment

The included studies were checked for the following risks of bias: publication bias (tested for by the Egger test), confirmation bias, halo/horn effect, primacy/recency bias, evaluative bias, cultural bias, social desirability bias, subjective judgement and lack of expertise related bias.

Effect measures: pooled correlation coefficients for psychometric scores related to assessment for autistic spectrum disorder between spouses were calculated where meta-analysis was appropriate and compared between spouses with offspring with and without a diagnosis of autistic spectrum disorder.

### 2.4. Synthesis Methods

Because of the heterogeneity of studies, the Hedges–Olkin random effects method was used for meta-analysis of intra-class correlation (ICC) and Spearman’s correlation coefficients.

The heterogeneity of studies was quantified and reported using Cochrane Q and I^2^ with values > 40% representing moderate heterogeneity, >60% substantial heterogeneity and >80% considerable heterogeneity.

If in separate studies, correlation of spousal’s mutual psychometric testing derived from the same cohort was investigated, only the investigation with the larger number of participants was included in the meta-analysis to avoid duplication.

To compare the pooled ICCs, Fisher’s z-transformation was used to convert the ICC coefficients into Fisher’s Z-transformed values followed by use of the following formula:

$$Zobs=\frac{Z1-Z2}{\sqrt{\frac{1}{N1-3}+\frac{1}{N2-3}}}$$

Z_obs_ = Fisher’s Z-statistic for the comparison. Z1 and Z2 are Fisher’s Z statistics of the ICCs of the two compared groups and N1 and N2 are the number of participants in each group.

For reporting bias assessment, both Begg–Mazumdar and Egger tests were applied to studies included in the meta-analysis.

Quality scores were compared between studies reporting on assortative mating of spouses with and without offspring with ASD using the Mann–Whitney U test.

We compared incidence rates (per 10,000 of population) of autism spectrum disorders in countries with generally predominant mating by individual choice as a more likely assortative mating strategy versus countries with other mating systems like those guided by arrangement by parents using chi-square testing and calculated the relative risk.

Statistical software used for meta-analysis was StatsDirect^®^ (STATSDIRECT LIMITED 36 Barton Hey Drive, Wirral, England, CH48 1PZ). Chi-square testing and risk ratio calculation was conducted using EpiInfo^®^ version 7.0 (CDC, Atlanta, GA, USA).

A *p*-value of <0.05 was taken as indicator of evidence of significantly low probability of erroneously rejecting the null hypothesis of no difference.

To calculate certainty assessment, we used the GRADE approach to certainty assessment, which included domains of risk of bias, inconsistency, imprecision, indirectness and publication bias [25].

For critical appraisal, we used the AXIS tool for assessment of the quality of cross-sectional studies using 20 items covering introduction, methods, results and discussion of findings [26].

If fully addressed by the authors, each item was attributed a scoring point with at least 70% of total equivalent to a high-quality publication, between 60 and 69% fair quality and below 60% low quality [27].

## 3. Results

### 3.1. Study Selection

**Figure 2:**
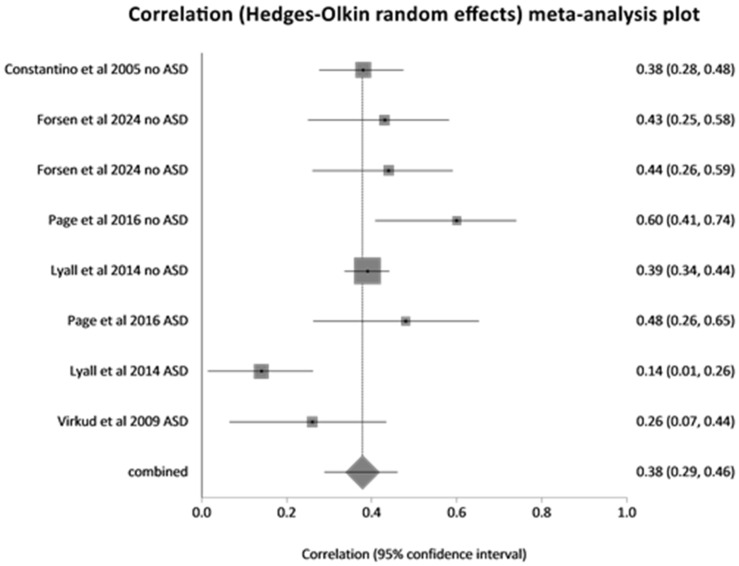
Meta-analysis of studies reporting intra-class correlation coefficients of spouse pairs’ social responsiveness scale scores for adults [28,29,30,31,32].

**Figure 3:**
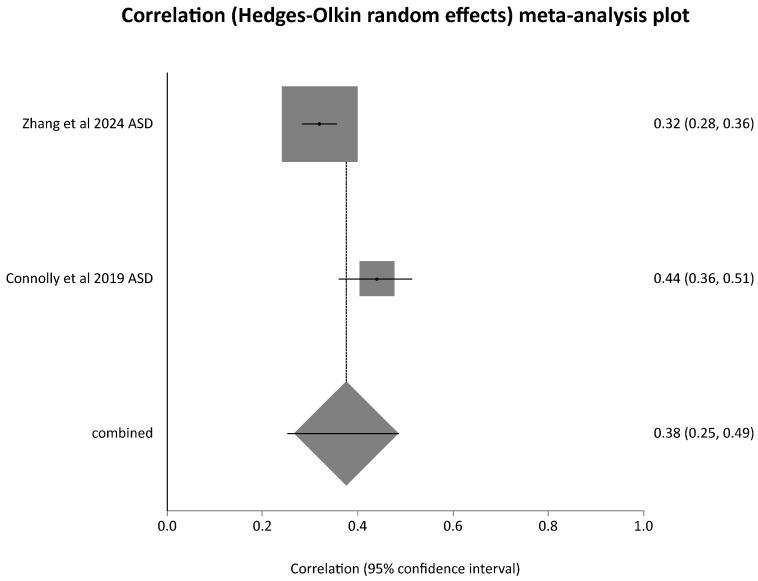
Meta-analysis of Spearman’s correlation coefficients of social responsiveness scale scores of spouses [33,34].

For studies on assortative mating, 14 investigations including 9914 spouse pairs, were included (see PRISMA Flow chart in Figure 1 for study selection and for content of studies Table 1). Table 1 and Table 2 provide information on study design, population and sample characteristics, comparisons, outcomes and key findings of each study. A critical analysis of quality using the AXIS tool and an analysis of limitation focusing on forms of bias detected were carried out. A total of 10 articles reporting on investigations into phenotypical parental assortative mating in 13 study groups totalling 6705 spouse pairs (see Table 1 and Table 2) were identified. In total, 10 studies (4641 spouse pairs) reported intra-class correlation (ICC) or Spearman’s correlation coefficients between spouses’ SRS scores. A meta-analysis could be conducted for eight study groups reporting intra-class correlation (ICC) between social responsiveness scale scores (SRSSs) (1927 spouse pairs) (see Figure 2) and separately on two studies (2674 spouse pairs) reporting Spearman’s correlation coefficients for SRSSs (see Figure 3). A further three studies could not be included in a meta-analysis as one reported on Pearson’s correlation coefficient of the Dutch Autism Spectrum Quotients (128 spouse pairs) and another on the Pearson’s correlation coefficients for SRSSs (121 spouse pairs) and a third on tetrachoric correlation of mating of spouses with a diagnosis of autism spectrum disorder (1855 spouse pairs). In two studies, correlation of subdomains of SRSS was investigated. Three articles reported on four study groups where genotypic assortative mating was investigated. Five studies detailed in Table 1 investigated correlation of parental social responsiveness scale score with severity of autism spectrum disorder in offspring. There were no studies identified which investigated the influence of mating comparing the severity of autism spectrum disorder in the offspring from spouses with similar versus dissimilar ethnic or ancestry related background.

**Table 1:** Characteristics of included studies regarding comparison of phenotype in parents of children with autistic spectrum disorder.

First Author and Year	Study Design	Population and Sample	Comparison	Outcome Measures	Key Findings	Quality Assessment Score (AXIS Tool)	Limitations
Constantino et al. 2005 [28]	Observational study	A total of 89 monozygotic female– female twin pairs and their parents, 69 dizygotic female–female twin pairs, 127 dizygotic male–female twin pairs and the parents of those twin pairs equivalent to 285 parent pairs. Age range from 8 to 17 years, with an average age of 12.5 years. European American, with 12.5% African American and <1% other ethnicity by self-report. The Missouri Twin Study population was the random sample from which study subjects were recruited.	Social responsiveness scale score (SRSS) compared between parents and parents and their children	Intraclass correlations of SRSS between parents and parents and their children. The mean SRS scores of the parents were compared to the mean SRS scores of the offspring.	Intraclass correlations for pairings of family members: mother–daughter 0.41, mother– son 0.38, father–daughter 0.49, father–son 0.58. Intraclass correlation between mothers and fathers was 0.38, *p* < 0.001 for all comparisons.	14	Bias forms associated with lay assessment ^1^ and there was a restriction of spousal reports to biological parents who were still married. This may have biased against the inclusion of parents with extreme scores (but may have protected from artificially inflated scores from disgruntled divorced parents).
Forsen et al. 2024 [29]	Observational study	Quantitative autistic trait data of parents of epidemiologically ascertained toddler twins were collected in the Early Quantitative Characterization of Reciprocal Social Behavior Study in Missouri with 95 spousal pairs and California with 93 spousal pairs. Birth records were used to identify all twin pairs born between 2011 and 2013 in those respective states, and parents were selected at random from pools of self-identified English-speaking Hispanic families in California and non-Hispanic white families in Missouri.	SRSS compared between parents	Intraclass correlation coefficients (ICCs) for square-root-transformed SRS-2 scores between spouses were calculated to index assortative mating. Quantile regressions tested the relationship between spousal SRS-2 scores based on estimates of regression coefficients at the 25th, 50th, and 75th quantile.	Missouri spousal pairs: intraclass correlation (ICC) of 0.43 (95% CI: 0.25, 0.58); California spousal pairs: 0.44 (95% CI: 0.26, 0.59. Quantile regression analysis showed that stronger associations between spousal SRS scores at higher quantiles. Significant spousal associations were observed at each quantile.	13	Bias forms associated with lay assessment ^1^
Zhang et al. 2024 [33]	Observational study	Family based autism collection: Simons Simplex Collection (SSC) including data on 2246 spouse pairs of European ancestry with one child with autism.	SRS obtained from an informant (mother reported on father and father reported on mother) 2246 spouse pairs, and the self-reported Broad Autism Phenotype Questionnaire (BAPQ) 2176 spouse pairs	The correlations between spouses’ measures of quantitative autistic traits in SSC were evaluated using Spearman’s correlation coefficient with subgroup analysis for parents of children with and without learning difficulties Comparison of the pattern of AM of autism w/and w/o CI/ID.	There was moderate and significant correlation of total scores and similar for children with and without learning difficulties (r = 0.32) for the SRS and weak with/without learning difficulties in children assessed by BAPQ: 0.12/0.15.	15	Bias forms associated with lay assessment ^1^ Selection bias ^2^
Smolen et al. 2023 [35]	Observational study	Family-based autism collection: Simons Simplex Collection (SSC) including data on 1822 spouse pairs of European ancestry with one child with autism.	SRS obtained from an informant (mother reported on father and father reported on mother), and the self-reported BAPQ	The correlations between spouses’ measures of quantitative autistic traits in SSC and were evaluated using Pearson’s correlation coefficient.	There was moderate significant correlation of total scores: r = 0.29 on SRS and 0.10 on BAPQ	12	Selection bias ^2^
Connolly et al. 2019 [34]	Observational study	Simons Simplex Collection (SSC) including data on 1953 spouse pairs of European ancestry with one child with autism.	SRS obtained from an informant (mother reported on father and father reported on mother)	The correlations between spouses’ measures of quantitative autistic traits in SSC were evaluated using Spearman’s correlation coefficient.	There was moderate significant correlation of total scores: r = 0.34 on SRS	14	Bias forms associated with lay assessment ^1^ Selection bias ^2^
Connolly et al. 2019 [34]	Observational study	Autism genome project database where data from 428 spouse pairs with 62% multiplex families (more than one child with autism)	SRS obtained from an informant (mother reported on father and father reported on mother)	The correlations between spouses’ measures of quantitative autistic traits in SSC were evaluated using Spearman’s correlation coefficient	There was a significant correlation of 0.44 for total scores observed.	14	Bias forms associated with lay assessment ^1^ Selection bias ^2^
Page et al. 2016 [30]	Observational study	A total of 151 Hispanic families recruited from the University of Miami Department of Psychology, categorized into two groups by index subject: ASD diagnosis (n = 85) and controls (n = 66) and their parents and siblings. The controls were families recruited from South Florida, including the University of Miami student community. In 79% of families, both parents were Hispanic.	Spanish version of SRS (see above) version 2 obtained from parents assessing each other. SRS-2 scores were obtained from children by parents and teacher report. All ASD subjects were diagnosed by expert clinicians at the University of Miami site; in addition to the SRS-2.	An intraclass correlation coefficient between parental SRS scores was calculated. Concordant elevation in parental scores of ASD subjects was analyzed comparing families with and without children with ASD by dividing the raw spouse-report SRS-2 data into four quartiles based on parental severity.	There was a significant correlation coefficient of 0.48 between parents of ASD families (62 spouse pairs) in ASD families and 0.60 in non-ASD families (60 spouse pairs)	16	Bias forms associated with lay assessment ^1^ Selection bias ^2^ The recruitment from a predominantly Hispanic background may have increased intraclass correlation due to common ancestors.
Lyall et al. 2014 [31]	Observational study	A total of 240 spouse pairs of children with autism spectrum disorder (maternal report of diagnosis) from the Nurses’ Health Study (NHS) II, a prospective cohort of 116 430 female nurses in the USA, with 92.2% with Caucasian ethnic origin. There were 993 control spouse pairs, which were parents with a child without a diagnosis of autistic spectrum disorder.	SRS-A was used and parents assessed each other. All indexed children were completed by the index child’s mother.	An intraclass correlation coefficient between parental SRS scores was calculated. The percentage of concordantly elevated parent score in offspring with ASD versus no ASD was reported.	Correlation of SRS-A scores between parents of children with ASD had a non-significant correlation coefficient of 0.14 and in the control group 0.39.	17	Bias forms associated with lay assessment ^1^ Selection bias ^2^
Van Steijn et al. 2012 [36]	Observational study	A total of 121 spouse pairs were recruited as part of the Biological Origins of Autism (BOA) project in the Netherlands which aims to examine the genetic, biochemical and cognitive origins of ASD and all children had a diagnosis of ASD.	Adult version of the autism spectrum quotient (AQ)	Correlation of the autism spectrum quotient using Pierson’s correlation coefficient	Both fathers and mothers scored above population average with respect to self-reported ASD symptoms but the correlation of symptom scores of the autism quotient was not significant.	16	The sample size calculation was small and the autism spectrum quotient data were from parental reports; therefore, they could not rule out outcome misclassification, inclusion of milder cases or other diagnoses.
Virkud et al. 2009 [32]	Observational study	Multiplex ASD families, n = 58, recruited from the Autism Genetic Resource Exchange and 2) 41 conservatively-defined simplex ASD families recruited for a longitudinal study of male sibling pairs at Washington University.	SRS-A was used and parents assessed each other. SRS scoring by one parent (in most cases, mother) and one teacher was completed.	An intraclass correlation coefficient between parental SRS scores was calculated. To compare spousal ICC correlation between families with one to families with more than one family member with ASD. To investigate whether concordantly elevated SRS scores in spouses are different in children of families with one compared to more than one member with ASD.	Correlation of SRS-A scores between parents of children with ASD had a significant (*p* < 0.01) correlation coefficient of 0.26 (99 spouse pairs).	14	Bias forms associated with lay assessment ^1^ Selection bias ^2^
Hoekstra et al. 2007 [37]	Observational study	Netherlands Twin Register kept by the Department of Biological Psychology at the VU University in Amsterdam. A total of 128 mother/father pairs were included. Participation rate for this data collection was 54%. Participating families did not significantly differ from non-participating families on socioeconomic status, but parental education level was slightly higher in participating families. No information about ASD diagnoses was available.	The parents completed the Dutch Autism-spectrum quotient (AQ) about each other.	The Pearson correlation coefficient between scores of spouses was calculated.	The partner correlation for AQ score was r = 0.05 (*p* = 0.59)	16	Participants were recruited on an informational day for parents of multiples. Individuals who dislike being confronted with large crowds may have been unlikely to attend this event.
Nordsletten et al. 2016 [38]	Observational study	Population-based cohort using Swedish population registers. Participants were all Swedish residents with a psychiatric diagnosis of interest along with their mates. A total of 880 men and their spouses and 975 women and their spouses were included.	Diagnosis (as determined by the treating physician). These diagnoses are documented using the World Health Organization’s International Statistical Classification of Diseases and Related Health Problems.	Tetrachoric correlation coefficient	ASD diagnosis correlated with a tetrachoric correlation coefficient of 0.48 for men and 0.45 for women which was highly significant (*p* < 0.01) and the highest for any psychiatric diagnosis and higher than the highest correlation coefficient for the control group with organic diagnoses.	16	The authors quoted detection bias as diagnosis in one spouse may have resulted in diagnosis in the spouse.

^1^ Confirmation bias, halo/horn effect, primacy/recency bias, evaluative bias, cultural bias, social desirability bias, subjective judgement and lack of expertise. ^2^ Inclusion of only families with an autistic child will lead to an increased probability of both patients to have autistic traits as it is an inherited condition.

**Table 2:** Characteristics of included studies regarding comparison of genotype in parents of children with autistic spectrum disorder.

First Author and Year	Study Design	Population and Sample	Comparison	Outcome Measures	Key Findings	Quality Assessment	Limitations
Zhang et al. 2024 [33]	Observational study	Family-based autism collections: the Simons Foundation Powering Autism Research for Knowledge (SPARK) (1575 families) and the Simons Simplex Collection (SSC) (2283 families). The reference ancestry population was a multi-ethnic population from the 1000 Genomes project. Autosomal SNPs only.	Polygenic risk scores (PGS) based on single nucleotide polymorphisms comparing both parents and compared to a reference ancestry population	Correlation between PGS of spouses	No correlation of PGS	16	The restriction to autosomal SNPs may have missed important alleles on the X-chromosome. Only the top and bottom 200 SNPs were analyzed.
Connolly et al. 2019 [34]	Observational study	A total of 1092 spouse pairs of the Autism Genome Project	Kinship coefficients and spousal correlation between the principal components using genome-wide single nucleotide polymorphism data on trio families.	Correlation between the principal components with comparison of the distribution of the kinship coefficients for spouses (mother/ father pairings) with the distribution of all other possible non-spouse pairings restricted to male/female pairings from the same ancestral background. The quantiles (from 0.001 to 0.999 in increments of 0.001) for the spouse pairs’ kinship coefficients were calculated and then mapped to the kinship coefficients for the non-spouse pairs; with the area under the deviation from a perfect correlation regression line (45 degrees indicating the degree of assortative mating.	Area under the curve 0.025 (95% CI 0.0110, 0.0389, indicating significant evidence of positive genetic assortative mating as spouses are more genetically similar than all possible nonspouse pairs	14	Only spouse pairs from the same ancestral population were included with similar proportions of ancestry to each other and to others in their population: The fact that this database included families with more than one member with autism may have increased the apparent assortative mating as parents who had both increased genetic burden would be over represented. The fact that all offspring had autism also increased apparent assortative mating for the same reason.
Connolly et al. 2019 [34]	Observational study	A total of 1221 spouse pairs of the Simons Simplex Collection data	Kinship coefficients and spousal correlation between the principal components using genome-wide single nucleotide polymorphism data on trio families.	See above from the same first author	Area under the curve was equal to −0.0062 (95% CI = −0.0187, 0.0065) indicating that there was no evidence of genetic assortative mating in the SSC data set.	14	The fact that all offspring had autism also increased apparent assortative mating for the same reason.
Weiner et al. 2017 [39]	Observational study	A total of 3209 spouse pairs of European ancestry from the Psychiatric Genomics Consortium Autism Group (PGC ASD) sample Autism Group from California, Montreal and Boston and Philadelphia, USA	Polygenic Risk Score based on common variant genotype data from the largest available independent Genome-Wide Association Study including effect sizes and *p*-values for each single nucleotide polymorphism (SNP) in the imputed GWAS analysis, typically using a *p*-value for SNP inclusion in PRS of 0.1.	Pearson correlation coefficient between maternal and paternal PRS	No correlation of PRS: r = 0.00050 with *p*-value of 0.78	5	An inappropriate high *p*-value for indication of significant SNPs was chosen, reducing the power of detection of a significant inter-spouse correlation.

### 3.2. Results of Syntheses

#### 3.2.1. Pooled Correlation of SRSS in Spouses

Meta-analysis of ICC of SRSS scores revealed a pooled correlation = 0.378 (95% CI = 0.289 to 0.461) which was statistically significant with a Z (test correlation differs from 0) = 7.76, *p* < 0.0001. This meta-analysis included both studies of families without an offspring with autistic spectrum disorder (ASD) (in Figure 1 designated as “no ASD” behind the year of publication) and those with an offspring with ASD (designated in the forest plot of Figure 1 as “ASD” behind the year of publication). This may account for some of the substantial heterogeneity detected which was reflected in a Cochran Q = 23.985 (df = 7) *p* = 0.0011, I^2^ (inconsistency) = 70.8% (95% CI = 23.3% to 84.2%). In a separate meta-analysis of studies reporting a Spearman’s correlation coefficient (both studies for families with offspring with ASD), a pooled correlation coefficient of 0.375 (95% CI = 0.252 to 0.487) was noted with a Z (test correlation differs from 0) = 5.648006 *p* < 0.0001. The heterogeneity of study results was considerable with a Cochran Q = 7.061587 (df = 1) *p* = 0.0079 and an I^2^ (inconsistency) = 85.8%.

#### 3.2.2. Correlation of Phenotype Subgroups of the Social Responsiveness Scales

In the two studies reporting on correlation of subdomains of the social responsiveness scales [33,34], there was no evidence for any of the subdomains for the SRS being differently correlated to others between spouse pairs.

#### 3.2.3. Correlation of Genotype Data in Parents of Children with Autistic Spectrum Disorder

Table 2 lists the results of studies detailing attempts at investigating evidence for correlation of genotypic characteristics of spouses of children with autistic spectrum disorder. Using polygenic risk scores for ASD derived from differences in single-nucleotide polymorphisms, no genetic evidence of assortative mating was found in three studies including 7067 spouse pairs with one autistic child [33,34,39]. One study group of 1092 spouse pairs of the Autism Genome project [34] demonstrated a significant spousal correlation between the principal components using genome-wide single nucleotide polymorphism data. The spouse pairs of this study had in 62% of cases more than one offspring with ASD as opposed to all the other studies, likely accounting for an increased burden of ASD-associated gene polymorphism in both spouses, and resulting in a significant correlation.

#### 3.2.4. Reporting Biases

**Figure 4:**
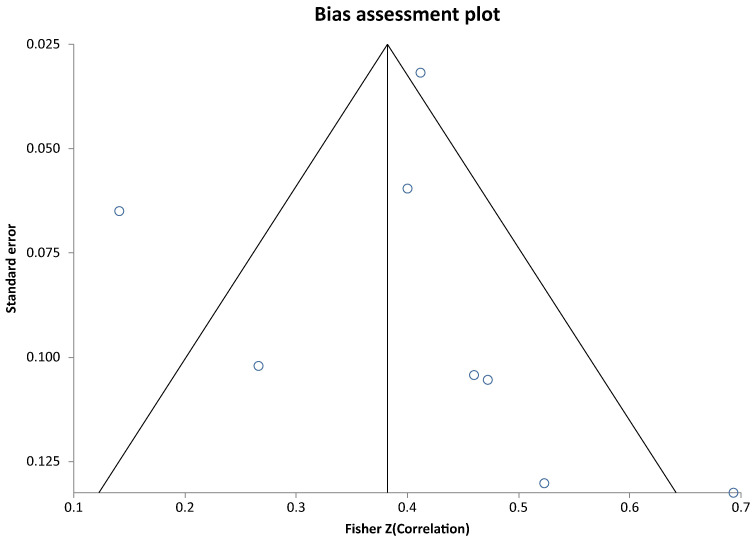
Fisher Z correlation score plotted against the respective standard error value.

The meta-analysis of the intra-class correlation coefficients analysis of reporting biases revealed a Begg–Mazumdar test: Kendall’s tau = 0.428571, *p* = 0.1789 and a non-significant Egger test result with a bias = 0.5240655053464733 (95% CI = −2.9882532201752032 to 4.0363842308681495) *p* = 0.7276 indicating that there was no evidence of reporting bias (see Bias assessment plot in Figure 4).

#### 3.2.5. Subgroup Analysis

Meta-analysis of intraclass correlation coefficients for social responsiveness scale scores (SRSSs) for study groups without known autistic spectrum disorders in parents or offspring (1526 spouse pairs) showed a pooled ICC of 0.40 (95% CI 0.35 to 0.46) with a Z-statistic of 13.42: *p* < 0.001. And for study groups with known autistic spectrum disorder in offspring (401 spouse pairs), they showed a pooled ICC of 0.278 (95% CI 0.08 to 0.46) with a Z-statistic of 2.723: *p* = 0.006. Comparison of ICC coefficients for SRSS between groups with and without ASD as offspring revealed a Z score of 190, indicating a highly significant difference: *p* < 0.0001 indicating a lower correlation of SRSS between parents of one or more children with autistic spectrum disorder compared to parents not chosen for having offspring with this disorder.

#### 3.2.6. Studies Investigating the Relationship of Parental Social Responsiveness Scale Scores with Autistic Spectrum Severity in Offspring

Five studies detailed in Table 1 [28,30,33,34,36] investigated correlation of parental social responsiveness scale score with severity of autism spectrum disorder in offspring. The key finding in the study of Lyall et al. [31] was that 12% of offspring with ASD versus 6% of controls had concordantly elevated parent scores (*p* < 0.001); liability to ASD was increased by approximately 90% among children with concordantly elevated parent scores (OR = 1.85, 95% CI 1.08, 3.16) and by approximately 50% when either parent’s score was elevated (OR = 1.52, 95% CI 1.11, 2.06). This finding was reproducible in the study by Constantino et al. [28] where the mean SRS scores for the offspring (*n* = 64 twins) of parents whose SRS scores both fell in the top quartile of their respective distributions (for parents in this sample) were substantially higher (by approximately 1.5 standard deviations) than those whose parents fell in any other SRS score quartile. Of these 64 children, 4 (from 4 different families) had SRS scores at or above 80 (i.e., within or above 1 standard deviation of the mean SRS score for children with PDD-NOS, in comparison to 3 such children (from 3 different families) in the entire remainder of the sample (*n* = 506 twins; Fisher’s exact *p* < 0.004). Only three of the parents of these clinical-level-affected offspring had SRS scores at or above 80. In another study [30], concordant elevations of mothers and fathers (i.e., both parents in upper quartile of the sample distribution) were observed more among ASD families than among non-ASD families (16.1 vs. 3.3%, Fisher’s exact, *p* = 0.03), and the former substantially exceeded the proportion expected by chance, which was 6.25%. These results were unchanged when restricting the analysis to data from one or the other specific language versions of the SRS-2 (Spanish vs. English). In the study by Virkud et al. [32], there was no significant difference in spousal correlations between families with a single child with ASD (SA) and families with multiple members affected by ASD (MA). Concordantly-elevated (upper quartile) spousal pairs were not significantly more common in MA versus SA families. In the study by Zhang et al. [33], there were no significant differences in the degree of spousal correlations for the quantitative autistic traits between groups with offspring with autism with versus without intellectual disability.

#### 3.2.7. Autism Prevalence in Countries with Assortative Mating Leading to Marriages Versus in Countries with Arranged Marriages

Table 3 lists studies of prevalence of autism spectrum disorder separate for countries known to have arranged marriages in the majority and countries with assortative mating determining marriages. Population-based studies on autism spectrum disorder prevalence were identified for the following countries with predominantly assortative mating determining marriage for the United States of America, Mexico, Venezuela, Aruba, The United Kingdom, France, Spain, Denmark, Poland, Sweden, Norway, Finland, Greece, Israel, Lebanon, Qatar, Oman, China, Taiwan, Australia and Faroe Islands including data on 7,072,330 participants with 44,660 identified with autistic spectrum disorder between the years 2008 and 2022, giving a pooled prevalence rate for autism spectrum disorder of 63.1 per 10,000 of the population in these countries.

Studies in countries with predominantly arranged marriages included data from India, Bangladesh, Sri Lanka, Nepal and Saudi Arabia from 111,297 participants with 157 diagnosed with autistic spectrum disorder resulting in a pooled prevalence rate for autistic spectrum disorder of 14.1 per 10,000 in those countries. The difference in prevalence rates was highly significant giving a risk ratio of autism with a culture of assortative mating versus one without of 4.4 (95% CI 3.8 to 5.2) (*p* < 0.0001).

#### 3.2.8. Certainty of Evidence

Regarding the certainty of the evidence for assortative mating, we came to the following conclusion: Across different populations in and outside the USA and using different analysis methods, there was evidence of significant assortative mating. The significant heterogeneity of study results indicates a degree of inconsistency and there were concerns about risk of bias as studies were based on self and spouses instead of expert report regarding social responsiveness scoring. Regarding phenotypical assortative mating, we judged the certainty of evidence therefore as low. Regarding genotypical assortative mating, we rated the certainty that there is no genetic assortative mating as very low because there is inconsistency, imprecision and indirectness of the evidence.

#### 3.2.9. Quality Assessment

Using the AXIS tool for assessment of all studies (n = 14) reporting on phenotypic assortative mating, 11 were of high, 2 of fair and one of low quality. Of the studies reporting on genotypic assortative mating, three were of high and one of low quality. Comparison of AXIS tool scores between studies included in the meta-analyses and with versus without offspring with ASD revealed a high median AXIS tool score of 14 for both types of studies without difference between groups (*p* = 0.643).

## 4. Discussion

### 4.1. General Interpretation

This systematic review demonstrated that there is assortative mating following social responsiveness scale scores in spouses with and without offspring with autistic spectrum disorder. The fact that the pooled correlation of scores with and without offspring with autistic spectrum was significantly different may reflect that in families without offspring with autistic spectrum disorder, there may have been a higher percentage of spouses who both scored their partner with low scores for autism-related items as they both had no features of autistic spectrum disorder, while spouses with offspring with autistic spectrum disorder may have both had increased scores consistent with autistic spectrum disorder in different domains of the scoring system reducing correlation. The genetic load causing autistic spectrum disorder will increase in the offspring if spouses have different domains affected, adding to the overall phenotypic severity in the offspring. Three studies which investigated the risk of increased SRS scores in offspring of both parents with correlating high SRS scores found higher SRS scores in offspring if both parents had more and significantly correlating autistic features. This illustrates that genetic load from both parents is additive in the offspring but was, as one study showed, not necessarily associated with more learning difficulties. The few data on subdomain correlation in social responsiveness scales between spouses did not show any particular domain correlating more than others but the number of studies was small.

Our results of a lower prevalence of autistic spectrum disorder in countries with a lower prevalence of assortative mating due to a dominance of arranged marriages were confirmed in the Global Burden of Disease study from 2021 on bigger data sets: In the subset comprising the largest data collections from countries with arranged marriages represented by the countries of South Asia which include Afghanistan, Bangladesh, Bhutan, India, Iran, Maldives, Nepal, Pakistan and Sri Lanka, the prevalence of autistic spectrum disorder was 68.6 per 10,000 (uncertainty interval 57.6 to 80.2), while in Western Europe and North America, it was 89.6 (uncertainty interval 75.1 to 105.4) and 109.7 (uncertainty interval 91.9 to 129.6), respectively [22]. The lack of denominators for the pooled prevalence data from the Global Burden of Disease Study made a statistical comparison of data impossible. The higher prevalence rates reported in the Global Burden of Disease Study compared to this systematic review may have been due to the fact that we included only carefully designed population-based studies using stringent criteria with validated tools in all participants.

### 4.2. Limitations

The social responsiveness scale was the tool most studies on assortative mating used, which is heavily weighted toward social impairments and most of the data in previous studies were generated among samples with IQ > 70. The SRS has not been evaluated systematically among populations with an intellectual disability [71]. The significant heterogeneity of study results may reflect bias as studies were based on self and spouses instead of expert report regarding social responsiveness scoring. Investigations into genotypical assortative mating were characterized by inconsistency, imprecision and indirectness of the evidence and there was a lack of ranking of genes according to degree of association with ASD and a lack of focus on investigating association of common high-impact genetic polymorphisms and lack of guidance by a sample size calculation providing adequate statistical power to detect significant correlations between spouses.

### 4.3. Implications for Practice, Policy and Future Research

Despite assortative mating offering an explanation for the increase in prevalence of autistic spectrum disorder, the rapid increase is difficult to understand in view of the fact that autism in the offspring reduces the number of further offspring within a family [72]. Future studies need to explore the dynamics of an increase in assortative mating promoted by information technology (e.g., internet facilitated dating), which enables distant people with “matching” personal characteristics to find each other. The impact of this assortative mating across distances needs to be investigated: For evolutionary survival of the genes generating a less reproductive phenotype, we hypothesize that there needs to be a protective gene constellation mitigating phenotypical features impairing reproduction which has co-evolved with genes encoding autistic spectrum disorder features within a specific group of people over a long period of history. This implies that offspring of spouses with a similar and elevated SRS score but different genetic (for example ethnic) background should have a higher risk of SRS score above both parents’ SRS score compared to offspring of spouses of the same genetic (ethnic) background. This is because the process of meiosis of the parental genes would entail a higher risk of losing one of the protective alleles evolved within a specific genetically more similar (e.g., ethnic) group. This hypothesis could be tested in future epidemiological and genetic studies. Investigations could hereby clarify whether assortative mating within a family or within a group of ethnically similar ancestry is associated with less severe autism in offspring compared to assortative mating between genetically less related families. Such research needs to correct for consanguineous unions (e.g., first cousins) which by themselves could lead to an increased risk of autosomal recessive gene mutations producing neurodevelopmental disorders with an autistic spectrum phenotype. There is an urgent need to investigate mating of which phenotypical features and which genes contributing to an autistic spectrum disorder phenotype are associated with learning difficulties in the offspring. Future research needs to investigate whether the findings of Nordsletten et al. [38] showing that people with clinically obvious autistic spectrum disorder are attracted to each other more than people with any other neurocognitive condition are reproducible in other ethnic groups without obstacles to assortative mating. If reproducible, the features guiding this attraction need to be defined. Preference for “sameness” and anxiety triggered by unpredictability of behavior are candidates to be explored.

Spousal assortment can occur on autistic spectrum disorder features without the persons having them being aware. To avoid severe autism in the offspring partners could be guided by improved informed choice provided through SRS scores which could be used to assess a future spouse similar to screening already taking place for conditions like thalassemia and others before starting a family.

## 5. Conclusions

There is evidence of assortative mating according to social responsiveness scale score which correlates significantly in spouse pairs with and without children with autistic spectrum disorder. In countries where assortative mating is predominant, a higher prevalence of autism spectrum disorder in children is found compared to countries without.

## Figures and Tables

**Table 3:** Characteristics of cross-sectional studies on autistic spectrum disorder prevalence with grouping into countries with and without mating of spouses by individual choice (assortative mating).

First Author and Year	Year of Publication	Country	Age of Screened Population (Years)	Diagnostic Tool	Incidence of Autism Spectrum Disorder per 10,000 of Population (Number of Children Assessed)
Countries with majority of mating by choice					
Shaw et al. 2025 [40]	2025	USA	8	Diagnosis as recorded in health and education data bases Overall, 66.5% of children aged 8 years with ASD had any documented autism test: ADOS ^1^ (39.6% overall), ASRS ^2^ (30.2% overall;), CARS ^3^ (24.1%), Gilliam Autism Rating Scale (12.2%), SRS ^4^ (12.0%) and ADI-R ^5^ (2.7%)	322 (274,857)
Van Balkom et al. 2009 [41]	2009	Aruba	0–9	DSM ^6^-IV	19 (13,109)
Chaaya et al. 2016 [42]	2016	Lebanon	1–10	DSM ^6^-IV	153 (998)
Raz et al. 2015 [43]	2015	Israel	8	M-CHAT ^7^	37 (2,431,649)
Chien et al. 2011 [44]	2011	Taiwan	17	DSM ^6^	28.7 (372,642)
Sun et al. 2019 [45]	2019	China	6–10	DSM ^6^ IVTR	97 (72,697)
Zhou et al. 2020 [46]	2020	China	6–12	DSM ^6^ IVTR	29 (125,c806)
Fombonne et al. 2016 [47]	2016	Mexico	8	Longitudinal ageing study in India	87 (4195)
Montiel-Nava et al. 2008 [48]	2008	Venezuela	3–9	ADOS ^1^	17 (254,905)
Alshaban et al. 2019 [49]	2019	Qatar	5–12	QSS-SCQ ^8^	930 (9074)
Al-Mamri et al. 2019 [50]	2019	Oman	0–14	DSM ^6^-5	20.35 (837,655)
Nygren et al. 2012 [51]	2012	Sweden	2	M-CHAT ^7^	80 (5007)
Idring et al. 2015 [52]	2015	Sweden	0–27	ICD ^9^-10	154 (735,096)
Morales-Hidalgo et al. 2018 [53]	2018	Spain	4–11	ADOS ^1^, ADI-R ^5^	150 (2765)
Fuentes J et al. 2021 [54]	2021	Spain	7–9	SCQ ^10^	59 (14,734)
Skonieczna-Zydecka et al. 2017 [55]	2017	Poland	0–16	ADOS ^1^, Q-CHA ^11^	35 (707,975)
Hansen et al. 2015 [56]	2015	Denmark	0–20	ICD ^9^-8 and ICD ^9^-10	59.2 (677,915)
Posserud et al. 2010 [57]	2010	Norway	7–9	ASSQ ^12^, DAWBA ^13^, DISCO ^14^	87 (6609)
Mattila et al. 2011 [58]	2011	Finland	8	ASSQ ^12^, ADOS ^1^, ADI-R ^5^, FSIQ ^15^	84 (4422)
Van Bakel et al. 2015 [11]	2015	France	7	ICD ^9^-10	36.5 (307,751)
Thomaidis et al. 2020 [59]	2020	Greece	10–11	ICD ^9^	115 (182,879)
Williams et al. 2008 [60]	2008	United Kingdom	11	DSM ^6^-IV	61.9 (14,062)
Randall et al. 2015 [61]	2015	Australia	6–7	DSM ^6^-5	196 (8400)
Kočovská et a. 2012 [62]	2012	Faroe Island	15–24	ASSQ ^12^, ADOS ^1^	94 (7128)
Countries with majority of mating determined by arrangement by parents or astrology					
Al-Zahrani et al. 2013 [63]	2013	Saudi Arabia	7–12	DSM ^6^, ASSQ ^12^	3.5 (22,950)
Akhter et al. 2018 [64]	2018	Bangladesh	1.5–3	CHAT ^7^	7.5 (5286)
Raina et al. 2017 [65]	2017	India	1–10	Indian scale for assessment of autism	15 (28,070)
Rudra et al. 2017 [66]	2017	India	3–8	Social and communication disorders checklist, ADOS ^1^, SCQ ^10^	23 (5947)
Poovathinal et al. 2016 [67]	2016	India	1–3	DSM ^6^	23.3 (18,480)
Nair et al. 2025 [68]	2025	India	0–108	CARS ^3^-2	1.5 (26,092)
Perera et al. 2009 [69]	2009	Sri Lanka	1.5–2	M-CHAT ^7^	740 (374)
Heys et al. 2018 [70]	2018	Nepal	9–13	Autism quotient-10	34 (4098)

^1^ Autism Diagnostic Observation Schedule, ^2^ Autism Spectrum Rating Scales, ^3^ Childhood Autism Rating Scale, ^4^ social responsiveness scale, ^5^ Autism Diagnostic Interview-Revised, ^6^ Diagnostic and Statistical Manual of Mental Disorders of the American Psychiatric Association, ^7^ modified checklist for autism in toddlers, ^8^ Qatar School survey social communication questionnaire, ^9^ International Classification of Diseases, ^10^ social communication questionnaire, ^11^ quantitative checklist for autism, ^12^ autism spectrum screening questionnaire, ^13^ development and wellbeing assessment, ^14^ diagnostic interview for social and communication disorders, ^15^ full-scale IQ.

## Data Availability

No new data were created or analyzed in this study.

## Appendix A

**Table A1:** PRISMA 2020 checklist ^1^.

Section and Topic	Item	Checklist Item	Location Where Item is Reported
**TITLE**			
Title	1	Identify the report as a systematic review.	Page 1
**ABSTRACT**			
Abstract	2	See the PRISMA 2020 for abstracts checklist.	Page 2
**INTRODUCTION**			
Rationale	3	Describe the rationale for the review in the context of existing knowledge.	Page 3
Objectives	4	Provide an explicit statement of the objective(s) or question(s) the review addresses.	Page 3
**METHODS**			
Eligibility criteria	5	Specify the inclusion and exclusion criteria for the review and how studies were grouped for the syntheses.	Page 3
Information sources	6	Specify all databases, registers, websites, organizations, reference lists and other sources searched or consulted to identify studies. Specify the date when each source was last searched or consulted.	Page 4
Search strategy	7	Present the full search strategies for all databases, registers and websites, including any filters and limits used.	Page 4
Selection process	8	Specify the methods used to decide whether a study met the inclusion criteria of the review, including how many reviewers screened each record and each report retrieved, whether they worked independently, and if applicable, details of automation tools used in the process.	Page 4
Data collection process	9	Specify the methods used to collect data from reports, including how many reviewers collected data from each report, whether they worked independently, any processes for obtaining or confirming data from study investigators, and if applicable, details of automation tools used in the process.	Page 4
Data items	10a	List and define all outcomes for which data were sought. Specify whether all results that were compatible with each outcome domain in each study were sought (e.g., for all measures, time points, analyses), and if not, the methods used to decide which results to collect.	Page 4
	10b	List and define all other variables for which data were sought (e.g., participant and intervention characteristics, funding sources). Describe any assumptions made about any missing or unclear information.	Page 4
Study risk of bias assessment	11	Specify the methods used to assess risk of bias in the included studies, including details of the tool(s) used, how many reviewers assessed each study and whether they worked independently, and if applicable, details of automation tools used in the process.	Page 5
Effect measures	12	Specify for each outcome the effect measure(s) (e.g., risk ratio, mean difference) used in the synthesis or presentation of results.	Page 5
Synthesis methods	13a	Describe the processes used to decide which studies were eligible for each synthesis (e.g., tabulating the study intervention characteristics and comparing against the planned groups for each synthesis (item #5)).	Page 5
	13b	Describe any methods required to prepare the data for presentation or synthesis, such as handling of missing summary statistics, or data conversions.	Page 5
	13c	Describe any methods used to tabulate or visually display results of individual studies and syntheses.	Page 5
	13d	Describe any methods used to synthesize results and provide a rationale for the choice(s). If meta-analysis was performed, describe the model(s), method(s) to identify the presence and extent of statistical heterogeneity, and software package(s) used.	Page 5
	13e	Describe any methods used to explore possible causes of heterogeneity among study results (e.g., subgroup analysis, meta-regression).	Page 5
	13f	Describe any sensitivity analyses conducted to assess robustness of the synthesized results.	Not applicable
Reporting bias assessment	14	Describe any methods used to assess risk of bias due to missing results in a synthesis (arising from reporting biases).	Page 5
Certainty assessment	15	Describe any methods used to assess certainty (or confidence) in the body of evidence for an outcome.	Page 5
**RESULTS**			
Study selection	16a	Describe the results of the search and selection process, from the number of records identified in the search to the number of studies included in the review, ideally using a flow diagram.	Page 4
	16b	Cite studies that might appear to meet the inclusion criteria, but which were excluded, and explain why they were excluded.	Not applicable
Study characteristics	17	Cite each included study and present its characteristics.	Page 4 to 20
Risk of bias in studies	18	Present assessments of risk of bias for each included study.	Page 4 to 20
Results of individual studies	19	For all outcomes, present, for each study: (a) summary statistics for each group (where appropriate) and (b) an effect estimate and its precision (e.g., confidence/credible interval), ideally using structured tables or plots.	Page 10, 11 and 14
Results of syntheses	20a	For each synthesis, briefly summarize the characteristics and risk of bias among contributing studies.	Page 6 to 15
	20b	Present results of all statistical syntheses conducted. If meta-analysis was done, present for each the summary estimate and its precision (e.g., confidence/credible interval) and measures of statistical heterogeneity. If comparing groups, describe the direction of the effect.	Page 10 and 11
	20c	Present results of all investigations of possible causes of heterogeneity among study results.	Page 14 and 19
	20d	Present results of all sensitivity analyses conducted to assess the robustness of the synthesized results.	Not applicable
Reporting biases	21	Present assessments of risk of bias due to missing results (arising from reporting biases) for each synthesis assessed.	Not applicable
Certainty of evidence	22	Present assessments of certainty (or confidence) in the body of evidence for each outcome assessed.	Page 19
**DISCUSSION**			
Discussion	23a	Provide a general interpretation of the results in the context of other evidence.	Page 19 and 20
	23b	Discuss any limitations of the evidence included in the review.	Page 20
	23c	Discuss any limitations of the review processes used.	Page 20
	23d	Discuss implications of the results for practice, policy, and future research.	Page 20
**OTHER INFORMATION**			
Registration and protocol	24a	Provide registration information for the review, including register name and registration number, or state that the review was not registered.	Page 21
	24b	Indicate where the review protocol can be accessed, or state that a protocol was not prepared.	Page 21
	24c	Describe and explain any amendments to information provided at registration or in the protocol.	Not applicable
Support	25	Describe sources of financial or non-financial support for the review, and the role of the funders or sponsors in the review.	Not applicable
Competing interests	26	Declare any competing interests of review authors.	Not applicable
Availability of data, code and other materials	27	Report which of the following are publicly available and where they can be found: template data collection forms; data extracted from included studies; data used for all analyses; analytic code; any other materials used in the review.	Not applicable

^1^ From reference [24].
